# Activation of TrkB with TAM-163 Results in Opposite Effects on Body Weight in Rodents and Non-Human Primates

**DOI:** 10.1371/journal.pone.0062616

**Published:** 2013-05-20

**Authors:** Mylène Perreault, Guo Feng, Sarah Will, Tiffany Gareski, David Kubasiak, Kimberly Marquette, Yulia Vugmeyster, Thaddeus J. Unger, Juli Jones, Ariful Qadri, Seung Hahm, Ying Sun, Cynthia M. Rohde, Raphael Zwijnenberg, Janet Paulsen, Ruth E. Gimeno

**Affiliations:** 1 Cardiovascular and Metabolic Diseases Research Unit, Pfizer Inc., Cambridge, Massachusetts, United States of America; 2 Global Biological Technologies (GBT), Pfizer Inc., Cambridge, Massachusetts, United States of America; 3 PDM, Pfizer Inc., Andover, Massachusetts, United States of America; 4 DSRD, Pfizer Inc., Andover, Massachusetts, United States of America; 5 Fort Dodge Animal Health, Pfizer Inc., Princeton, New Jersey, United States of America; Biological Research Centre of the Hungarian Academy of Sciences, Hungary

## Abstract

Strong genetic data link the Tyrosine kinase receptor B (TrkB) and its major endogenous ligand brain-derived neurotrophic factor (BDNF) to the regulation of energy homeostasis, with loss-of-function mutations in either gene causing severe obesity in both mice and humans. It has previously been reported that peripheral administration of the endogenous TrkB agonist ligand neurotrophin-4 (NT-4) profoundly decreases food intake and body weight in rodents, while paradoxically increasing these same parameters in monkeys. We generated a humanized TrkB agonist antibody, TAM-163, and characterized its therapeutic potential in several models of type 2 diabetes and obesity. In vitro, TAM-163 bound to human and rodent TrkB with high affinity, activated all aspects of the TrkB signaling cascade and induced TrkB internalization and degradation in a manner similar to BDNF. In vivo, peripheral administration of TAM-163 decreased food intake and/or body weight in mice, rats, hamsters, and dogs, but increased food intake and body weight in monkeys. The magnitude of weight change was similar in rodents and non-human primates, occurred at doses where there was no appreciable penetration into deep structures of the brain, and could not be explained by differences in exposures between species. Rather, peripherally administered TAM-163 localized to areas in the hypothalamus and the brain stem located outside the blood-brain barrier in a similar manner between rodents and non-human primates, suggesting differences in neuroanatomy across species. Our data demonstrate that a TrkB agonist antibody, administered peripherally, causes species-dependent effects on body weight similar to the endogenous TrkB ligand NT-4. The possible clinical utility of TrkB agonism in treating weight regulatory disorder, such as obesity or cachexia, will require evaluation in man.

## Introduction

Obesity is a debilitating disorder associated with several co-morbidities, including type 2 diabetes and cardiovascular disease. It is well recognized that a tight regulation of the balance between energy intake and energy expenditure is key for weight neutrality, and numerous factors have been involved in this highly regulated and conserved process. Recently, the neurotrophin family of growth factors, more specifically brain-derived neurotrophic factor (BDNF) and neurotrophin-4 (NT-4) has been implicated in the regulation of energy balance. Loss-of-function mutations in BDNF or its receptor, tyrosine receptor kinase B (TrkB), have been associated with severe obesity and hyperphagia in both humans and mice [1]–[5], and studies in mice have shown that ablation of BDNF specifically in neurons is sufficient to induce obesity [6]. Central administration of BDNF or NT4 decreased food intake in mice and non-human primates (NHPs) at relatively low concentrations, suggesting that neurotrophins can regulate food intake by activating TrkB in deeper brain structures [7], [8]. Consistent with these findings, peripheral BDNF or NT-4 administration induced body weight loss in several rodent models of obesity and diabetes, and the effect was mainly caused by appetite suppression [9], [10]. However, in contrast to rodents, peripheral injection of the TrkB ligand NT-4 resulted in a paradoxical increase in food intake and body weight in lean and obese NHPs [7], suggesting different mechanisms of TrkB activation between rodents and NHPs.

In rodents and humans, TrkB and BDNF are highly expressed in two major appetite-regulatory centers: the hypothalamus (HT) and the dorsal vagal complex of the brain stem (DVC) [11]–[13]. BDNF injections directly into the HT or DVC resulted in significant decreases in food intake and body weight, suggesting that BDNF can act at multiple appetite-regulatory sites [8], [11]. It is well recognized that the central nervous system is protected by the blood brain barrier (BBB), which creates tight junctions around the capillaries and prevents the entry of large molecules into the brain. However, specialized regions of the CNS positioned near the ventricular system and called circumventricular organs (CVOs) contain fenestrated endothelia rather than tight junctions and allow access of large molecules to structures, including the median eminence located near the arcuate nucleus (ARC) of the HT and the area postrema (AP) which constitutes part of the DVC [14]. It is well documented that peripherally injected appetite-regulatory antibodies can localize to these sites, and their body weight regulatory effects are thought to be mediated through access to CVOs [15], [16]. TrkB ligands may also act through these sites, and differences in the permeability or microanatomical location of the BBB in these regions between rodents and NHPs could possibly explain the reported food intake and body weight differences after peripheral injections.

In addition to TrkB, the endogenous TrkB ligands BDNF and NT-4 both bind to and activate a second structurally unrelated neurotrophin receptor, p75NTR. Activation of p75NTR induces cellular responses that are often the opposite of TrkB activation. For example, activation of p75NTR by neurotrophins promotes apoptosis rather than cell survival and facilitates hippocampal long-term depression rather than long-term potentiation [17]. Unlike neurotrophins, TrkB agonist antibodies do not recognize p75NTR, and thus are more specific TrkB reagents that allow examination of the effects of TrkB activation in the absence of p75NTR [18].

To evaluate the therapeutic potential of activating TrkB *in vivo* and to understand better the species differences and mechanisms behind them, we have generated a fully humanized TrkB agonist antibody, TAM-163, and tested its efficacy in several preclinical species and in different models of obesity. Our results confirm the previous observation that peripheral TrkB activation causes opposite effects on body weight and food intake in rodents and non-human primates and suggest that the mechanism is independent from differences in brain penetration between species.

## Materials and Methods

### Protein Generation

TAM-163 is a humanized derivative of 29D7 [18] containing the human IgG1 heavy chain and the human kappa light chain. The human IgG1 control protein consists of the variable domains of a human anti-tetanus toxin antibody (obtained from ATCC) fused to the constant regions of human IgG1. Unless otherwise indicated, control proteins and TrkB extracellular domain recombinant proteins were obtained from R&D Systems. An expression vector containing humanized (human IgG1) 29D7, designated TAM-163, was stably transfected into CHO cells. Supernatant was collected from cells grown in serum-free medium, and TAM-163 was purified by Protein A affinity chromatography followed by size exclusion chromatography. The final protein preparation was >99% pure, and was stored at 4°C in 10 mM L-Histidine-5%sucrose (his-sucrose), pH 5.5–6.5 buffer.

### Surface Plasmon Resonance (SPR)

Human and mouse TrkB extracellular domain recombinant proteins were immobilized at a low density on a CM5 chip (41 and 30 response units respectively) and various concentrations of TAM-163 were injected over the surface using a 2 minute association and 4 minute dissociation. The surface was regenerated with 4 M MgCl_2_ between injection cycles. Data analysis was done using a 1∶1 fit model and the affinity constants ka (association rate constant), kd (dissociation rate constant) and KD (affinity) were calculated.

### Generation of hTrkA-Cre, hTrkB-Cre and hTrkC-Cre Cell Lines

The hTrkA-Cre, hTrkB-Cre and hTrkC-Cre cell lines have been described before [18]. To generate a mouse TrkB-Cre cell line, the full-length mouse TrkB open reading frame was subcloned into pcDNA3.1-Hyg (Invitrogen) and transfected into HEK293 cells stably expressing a luciferase reporter construct. Stable clones were isolated and screened for expression of TrkB by Western blotting, for signaling by Cre-luciferase reporter assay as well as TrkB and ERK1/2 phosphorylation. Clones with moderate expression of TrkB were found to have the best signal to background ratio in signaling assays and one representative clone was selected for signaling experiments. A cell line stably expressing dog TrkB was generated in HEK293 cells. Cells expressing intermediate levels of dog TrkB were identified by Western blotting using anti-Trk antibodies (Becton Dickinson and Chemicon) and individual clones were tested for their ability to mediate BDNF-induced phosphorylation events.

### Cre-luciferase Reporter Assays

Stable cell lines expressing both a CRE-luciferase reporter construct and hTrkA (hTrkA-Cre), hTrkB (hTrkB-Cre) or hTrkC (hTrkC-Cre) were generated using standard techniques [18], [19]. Cells were plated at 35,000 cells/well in 100 µL growth medium (DMEM, 10% FCS) in 96-well plates. The next day, 10 µL of antibody or endogenous ligand was added to each well and cells were incubated for another 16–18 hours. Luciferase activity was measured using the Steady-Glo Luciferase Assay System (Promega, E2520) according to the manufacturer’s protocol.

### SHC1 Recruitment Assay

SHC1 recruitment to phosphorylated hTrkA, hTrkB, and hTrkC was measured using the Pathhunter technology (Discoverx). 10,000 cells/well were seeded in a 384-well plate in 20 µl MEM +0.5% horse serum, and incubated overnight at 37°C. 5 µL/well of agonist or control was added at the designated concentration, and plates were incubated at room temperature for 3 hours. Then 12 µL/well of enzyme substrate (PathHunter Detection Reagent Mix, Discoverx) was added, and further incubated for one hour before reading the chemiluminescent signal in the EnVision (PerkinElmer). Experiments were performed in triplicate.

### Detection of Phosphorylation by Western Blotting

5×10e5 cells/well in DMEM, 10% FBS, were plated in 6-well plates and grown to 85–90% confluency. Cells were washed once with DMEM, 0.1% FBS and then incubated in the same medium for 4 hours. Cells were treated with antibodies or endogenous ligands for 15–60 minutes. Cells were lysed and lysates were heated at 100°C for 5 min. Samples were resolved on a NuPAGE 4–20% Bis-Tris gradient gel (Invitrogen) and Western blotting was performed using anti-phospho-Trk (Tyr490), anti-phospho-PLCγ1 (Tyr783), anti-phospho-AKT (Ser473), anti phospho-ERK1/2 (p44/p42 Thr202/Tyr204) from Cell Signaling or anti-actin (Sigma). Membranes were incubated with the appropriate HRP-conjugated secondary antibody (Cell Signaling) for 2 hours. The signals were developed using the ECL kit (GE Healthcare RPN2106V) followed by X-ray film exposure or Gel-Doc (Bio-Rad) to capture the image.

TAM-163-mediated signaling was assessed in human neuroblastoma (SH-SY5Y) cells as follows. SH-SY5Y cells were obtained from ATCC (CRL-2266) and were maintained in DMEM:F12 (1∶1) growth medium supplemented with 2 mM L-glutamine, 15% fetal bovine serum (FBS), 100 U/mL penicillin and 100 µg/mL streptomycin. Cells were incubated with 10 µM all-trans retinoic acid (BioMol International GR-100) for 3 days to induce differentiation. Cells were then cultured in low serum media (growth media with 1% FBS) overnight, and further cultured in 0.1% FBS medium for 4 hours prior to addition of TAM-163. Cell lysis and western blotting was performed as described above.

### TrkB Internalization and Degradation

The TrkB internalization was detected by measuring cell surface associated TrkB in the presence or absence of TrkB activation as described [20]. hTrkB-Cre and SH-SY5Y cells were grown to 90% confluency, serum starved in 0.1% FBS for 4 hours, and then treated with 20 nM BDNF (R&D), 100 nM TAM-163 or 100 nM hIgG for the indicated times at 37°C to allow binding and internalization to occur. Cells were then put on ice and remaining cell surface proteins were biotinylated with 300 µg/mL Sulfo-NHS-Biotin (Thermo Scientific) for 30 minutes on ice. After quenching with 100 mM glycine (Sigma) and washing with cold PBS, cells were lysed (Lysis buffer: 0.1% SDS, 1% NP-40, 50 mM HEPES pH 7.4, 2 mM EDTA, 100 mM NaCl and protease inhibitors), and debris was cleared by centrifugation in an Eppendorf microfuge at 13,000 rpm for 10 minutes. Biotinylated proteins in the supernatants were allowed to bind to streptavidin-coated beads (Invitrogen, Dynabeads M-280 Streptavidin) for 1 hour at 4°C; beads were then washed twice with PBS, and protein was eluted by boiling beads with 40 uL of sample loading buffer (Invitrogen). Proteins were analyzed by western blotting using the following antibodies: anti-TrkB (Becton Dickinson), anti-EGF-receptor (Cell Signaling), and anti NMDA-receptor1 (Cell Signaling).

Degradation of the internalized TrkB was detected following the process as described [21]. hTrkB-Cre and SH-SY5Y cells were grown to 90% confluency and serum starved in 0.1% FBS for 4 hours. Cell surface proteins were biotinylated with 300 ug/mL Sulfo-NHS-Biotin (Thermo Scientific) for 30 min on ice. Cells were treated with 10 nM BDNF, 100 nM TAM-163, or 100 nM hIgG1 for the indicated time. Cells were lysed and biotin labeled TkB was processed and analyzed by western blot as described above.

### Cloning and Sequencing of TrkB Across Several Species

Human TrkA (hTrkA), human TrkB (hTrkB), human TrkC (hTrkC), and mouse TrkB (mTrkB) cDNAs were cloned into the mammalian expression vector pcDNA3.1-Hyg (Invitrogen) using standard molecular cloning techniques. Human p75NTR was cloned into the mammalian expression vector pSMED2 (Pfizer internal vector). The final constructs were confirmed by sequencing; the open reading frame was found to be 100% identical to NM_002529.3, NM_006180, NM_001012338.1, NM_001025074 and NM_002507 for hTrkA, hTrkB, hTrkC, mTrkB and hp75NTR, respectively. TrkB from cynomolgus monkey, and dog was cloned by PCR using the Stratagene Easy-A High-Fidelity system (Stratagene, La Jolla, CA) with brain cDNA from each species as a template (BioChain, Hayward, CA, USA), and the following primers: Cynomolgus monkey:5′GAATTCGCCGCCACCATGTCGTCCTGGATAAGGTGGCATGGAC and 5′GCGGCCGCCTAGCCTAGAATGTCCAGGTAGACCGGAG; Dog: 5′GGATCCGCCGCCACCAT-GTCGTCCTGGACGAGGTGGCATGG and 5′GCGGCCGCCTAGCCTAGAATATCCAGGTAGAC-TGGA. PCR fragments were cloned into the pCR2.1-TOPO and pcDNA3.1-Hyg vector using standard molecular cloning techniques, and 8–16 clones per species were sequenced. For cynomolgus monkeys, among 10 clones sequenced, 2 TrkB-a and 8 TrkB-c splice variants were identified, and a total of 23 nucleotide polymorphisms (20 silent, 3 missense) were noted. These polymorphisms could reflect the outbred nature of the monkey population, but could also be due to PCR artifacts. The most commonly occurring sequence variants (TrkB-c splice variant) were used for sequence comparisons and to generate expression constructs.

### Animal Studies

All animals were housed in a temperature-controlled (∼25°C), 12-hour light/dark cycle facility and were fed chow or high-fat diets and water *ad libitum* unless otherwise indicated. For all in vivo studies, the dose was based on the most recent scheduled body weights recorded prior to dosing.

### Ethics Statement

All experimental work was conducted in accordance with the humane guidelines for ethical and sensitive care by the Institutional Animal Care and Use Committee (IACUC) of the US National Institutes of Health. All studies conducted in our facilities were performed under Pfizer IACUC approved protocols. Study performed in male obese rhesus monkeys was approved by the Charles River Laboratory IACUC. Specific steps to monitor and alleviate discomfort associated with the procedures are described below.

### Efficacy of TAM-163 in Animals

Six-week-old male C57BL/6J mice were fed a high-fat diet (D12492, Research Diets) for 11 weeks at The Jackson Laboratory (Bar Harbor, ME, USA) and acclimated one per cage in our facility for an additional 2 weeks before study initiation. Mice received 3 intraperitoneal (IP) doses of IgG control (10 mg/kg in PBS) or TAM-163 (1, 3 and 10 mg/kg in PBS) on days 1, 8 and 15. Body weight and food intake were measured weekly. Animals were euthanized by CO_2_ asphyxiation 22 days after the first dose, blood was collected and serum extracted for insulin measurements.

Twelve-week-old male Zucker Diabetic Fatty (ZDF) rats were purchased from Charles River (Wilmington, MA) and housed one per cage in our facility for 3 weeks prior to study initiation. Animals received 2 doses of IgG control (10 mg/kg in PBS, IP) or TAM-163 (0.1, 1 and 10 mg/kg in PBS, IP) on days 1 and 4. Body weight and food intake were measured every other day. Animals were euthanized by CO_2_ asphyxiation 7 days after their first dose, blood was collected and serum extracted for insulin measurements.

Ten to thirteen-week old male Syrian Gold hamsters were obtained from Harlan (South Easton, Ma), housed 3 per cage and acclimated in our facility for one week on standard chow diet followed by two weeks on high-fat high-cholesterol diet before study initiation. During the last 2 weeks of acclimation and throughout the study, hamsters received 10% sucrose in their drinking water. Animals were dosed twice with IgG control (10 mg/kg, IP) or TAM-163 (3 and 10 mg/kg, IP) in his-sucrose vehicle (10 mM L-Histidine 5% Sucrose pH 5.5–6.5) on days 1 and 8. Body weight was measured weekly. Animals were sacrificed by CO_2_ asphyxiation at the end of the study.

Adult beagle dogs from an in-house colony were singly housed and fully acclimated for at least 6 weeks with access to regular chow diet once a day. Animals received a single dose of vehicle (his-sucrose, SC) or TAM-163 (10 mg/kg, SC) on day 1. Body weight was recorded twice weekly for the first eight weeks of the study (until day 28), and once weekly thereafter. After completion of the experimental phase of the study, dogs were returned to the Pfizer Animal Health colony.

Three to six year-old male rhesus monkeys were fed a high-fat diet (45% kcal fat D06051301A, Research Diets) for 9 months prior to study initiation and housed individually in stainless steel cages. The study was conducted at Charles River Laboratory (Shrewsbury MA). Animals were acclimated to pole/collar and chair before the performance of the required technical procedures. General health as well as moribundity/mortality checks were performed twice daily (AM and PM) throughout the course of the study. Animals were trained to feed for 1.5–2.5 hours twice daily (minimum of 6 hours apart) approximately 2 weeks before the start of the study to minimize spillage and to acclimate them to a consistent feeding regimen. Animals were given at least 260 g of food at each feeding which was quantitatively verified by weighing. After animals were allowed to feed for the 1.5–2.5-hours duration, the amount of food consumed was determined by weighing the remaining uneaten food. If there was excessive spillage, or other related observations that could impact the food consumption measured, this was documented in the study file. For each feeding, the uneaten food was discarded and new food provided. As part of the primate enrichment program, diet was supplemented with washed, fresh produce once daily at one of the feedings and animal commingling was allowed when food consumption or behavioral assessments were not being monitored. In the single dose study, animals received 4 weekly doses of vehicle (his-sucrose, IV) followed by a single dose of TAM-163 (1, 3 or 10 mg/kg, IV). Body weight was measured twice weekly while food intake was measured 5 days a week. For the multiple dose study, animals received 4 weekly doses of vehicle (his-sucrose, IV), followed by 4 weekly doses of TAM-163 (10 mg/kg, IV) and then received 4 additional weekly doses of vehicle. Body composition (described below) was determined only in the multiple dose study, and measurements were performed during the last week of initial vehicle and TAM-163 treatments. After completion of the experimental phase of the study, monkeys were returned to the Charles River Laboratory colony.

### Body Composition

Body composition was measured in male rhesus obese monkeys by Dual-Energy X-Ray Absorptiometry (DEXA) while the animals were tranquilized with either ketamine HCl (10 mg/kg, IM, to effect), or Telazol^®^ (2–6 mg/kg, IM, to effect). Analysis was performed using the General Electric (GE) Lunar DEXA according to the manufacturer’s operating instructions.

### Serum Insulin

Serum insulin was determined using the ultrasensitive rat insulin ELISA (Crystal Chem, Inc., Downers Grove, IL, USA).

### Pharmacokinetic/pharmacodynamic Studies in Lean Animals

For pharmacology studies in lean rats, 9 week-old male and female Sprague-Dawley rats were obtained from Charles River (St-Constant, Quebec, Canada) and acclimated in our facility before receiving 2 doses of TAM-163 at 3 mg/kg via a jugular vein catheter or a single intravenous (IV) bolus dose at 20, 60 or 200 mg/kg. Body weight measurements and serum collection for determination of test article concentration by an immunoassay were conducted at pre-dose and after 28 days for the 3 mg/kg dose group or pre-dose and periodically through 24 days for the 20, 60 and 200 mg/kg groups. Animals were sacrificed by CO_2_ asphyxiation at the end of the experimental procedures.

Naïve 3–6 year-old male and female cynomolgus lean monkeys were housed in our facility and given a daily food ration of Teklad certified Global Primate Diet biscuits (with additional biscuits offered if all were consumed at several times throughout the day) and allowed water ad libitum. Care was taken that animals had excess biscuits for each meal. Fruits and vitamin supplements were also provided daily. General health as well as moribundity/mortality checks were performed at least once or twice daily, respectively, throughout the course of the study. Animals received a single IV bolus dose of TAM-163 (1, 3, 20, 60 or 200 mg/kg). Body weights were measured 7 days post dose for animals dosed at 1 and 3 mg/kg, or twice prior to dosing and twice weekly through 6 days postdose for females and through 24 days postdose for males dosed at 20, 60 and 200 mg/kg. Blood was collected and serum processed for measurement of TAM-163 concentrations periodically through 7 days postdose for females and through 24 days postdose for males dosed at 20, 60 and 200 mg/kg. At the end of the study, all monkeys were given glycopyrrolate with ketamine hydrochloride, anesthetized with a sodium pentobarbital solution and exsanguinated.

### Alexa Labeling of Antibody

For mice, Alexa labeling of TAM-163 or human IgG control was done using an Alexa Fluor 594 Protein Labeling Kit (Molecular Probes). First, 50 µL of cold 1 M sodium bicarbonate (Component B, pH 8.3, made in deionized water (dH2O)) was added to 500 µL of TAM-163 or human IgG control (2 mg/mL) and allowed to warm to room temperature (RT). The RT reaction was added to a RT vial of reactive dye included in the kit and reaction mixtures were stirred for 1 hour at RT. Labeled protein was purified by column filtration as described in the kit instructions and stored at 4°C. Degree of labeling was quantitated by measuring the absorbance of the conjugate solution at 290 nm and 590 nm in a cuvette with a 1 cm pathlength. Protein concentration (M) was calculated by [(A280–(A590×0.56))×dilution factor]/203,000 and degree of labeling (moles dye per mole protein) was calculated by (A590×dilution factor)/(7300,protein concentration (M). The dilution factor was 1∶100. Alexa labeled TAM-163 (Alexa-TAM-163) or Alexa labeled IgG control (Alexa-IgG) was diluted in vehicle (PBS) just prior to administration. The protein concentration was 15.66 mg/ml and administered within 1–2 days of production.

For NHPs, TAM-163 was thawed, formulated into PBS pH 7.2, concentrated to 20 mg/mL, and adjusted to 0.1 M NaHBCO3 pH 8.6. The Invitrogen Alexa Fluoro568 (A20103) was reconstituted with DMSO to 10 mg/mL, and was slowly added to the prepared TAM-163 solution. The reaction was incubated one hour at room temperature with continuous stirring. The reaction was stopped by formulation into PBS pH 7.2 via Sephadex G25 column. The peak was collected and analyzed by with OD280/577, Endotoxin LAL Assay, SEC-HPLC, SDS-PAGE, and IEF-PAGE. Alexa-labeled TAM-163 or IgG control was intravenously (IV) administered to monkeys at a volume of 1 mL/kg body weight.

### Animal Studies for Alexa-labeled TAM-163 Detection

Twelve adult (∼20 week old) male DIO C57BL/6NTac mice (28–50 g; Taconic Laboratories) were maintained one per cage in our facility and allowed high-fat diet (Research Diets, D12492, 60% HFD) and water *ad libitum* in a light- and temperature-controlled environment. Animals were weighed, assigned to 4 groups based on body weight and subsequently injected with Alexa-TAM-163 or Alexa-IgG (10 mg/kg, IP). Half the animals (n = 3 per group) were used to confirm that Alexa-labeling of TAM-163 did not affect its efficacy (acute food intake and body weight measurements). The other half was perfused 6 hours post-injection (n = 3/group). For procurement of tissue for histological examination, animals were deeply anesthetized with an IP injection of ketamine:xylazine (100∶20 mg/kg, IP) and subsequently perfused transcardially with 20 ml of 0.9% PBS followed by 20 mL of 4% paraformaldehyde. Brains were removed, stored in the same fixative overnight at 4°C, immersed in 30% sucrose in PBS, pH 7.0, at 4°C overnight, and cut on a cryostat coronally into six equal series at 30 µm. The sections were placed at 4°C in PBS until further histological processing.

Eight 12 to 15 year-old male cynomolgus monkeys (macaca fascicularis) were maintained in our facility and fed Harlan Primate Chow 2050 with water available *ad libitum* in light- and temperature-controlled environment. Animals were either singly housed or paired in one cage. Monkeys were given various produce such as apples, oranges, bananas, grapes, and peanuts. In addition, animals were supplied with various toys and fleece boards for enrichment. Animals were tranquilized with ketamine (10 mg/kg, IM) and injected with Alexa-TAM-163 or Alexa-IgG (10 mg/kg, IV) with n = 4 per group. Approximately 6 hours later, animals were administered Telazol (4 mg/kg, IM) followed by 5000 units of heparin and then deeply anesthetized with sodium pentobarbital (100 mg/kg, IV) and subsequently perfused transcardially with a saline solution (0.9% saline) at a perfusion rate of 2 L in 3–5 minutes. Animals were perfused until no red blood cells were evident exiting the right atrium. Next, animals were perfused with 6 L of 4% paraformaldehyde at the rate of 2 L per 15 minutes. Brains were extracted and blocked into anatomically relevant divisions. After blocking brain tissue, sections were post fixed in 4% paraformaldehyde for 2 hours at 4°C then transferred to 10% glycerol cyroprotectant for 24 hours at 4°C. Tissue blocks were then transferred to 20% glycerol cryoprotecant for 72 hours at 4°C. Tissue blocks were then flash frozen in a small beaker of isopentane on dry ice and placed in labeled foil packets and stored at -80°C. Tissue blocks were coronally sectioned on a cryostat into 24 equal series at 30 µm. The sections were placed at 4°C in PBS until further histological processing.

### Immunohistochemistry

For visualization of Alexa-TAM-163 labeling, free-floating sections of mouse or NHP tissues were mounted on SuperFrost Plus slides and coverslipped with vectashield hardest mounting medium with Dapi counterstain (Vector Laboratories). Due to the high level of auto-florescence in NHP tissue, anti-human IgG antibody coupled with a chromogenic detection was performed. Tissue was first rinsed in 1X PBS and then incubated in 3% H_2_0_2_ for 15 minutes to quench endogenous peroxidase activity. After washing, DNA denaturation was conducted by steaming sections in citrate buffer (0.1 M sodium citrate and 0.1 M citric acid pH 6.0) for 10 minutes, followed by washing in 1X PBS. Next, tissue was incubated in 2.0 N HCl for 30 minutes at RT then washed in PBS. Sections were blocked with 5% normal goat serum in 1X PBS for 30 minutes at room temperature and then incubated in anti-human IgG (1∶200, Sigma) for 3 days at 4°C. The IgG signal was further amplified with an avidin-biotin-peroxidase complex (Vector Laboratories, Burlingame, CA), followed by incubation with DAB (DAKO)+Nickel ammonium sulfate (Sigma) substrate. Tissue was mounted on SuperFrost Plus slides and coverslipped with Cytoseal XYL mounting medium.

### Data Analysis and Production of Photomicrographs

Antibody binding patterns were viewed with a Nikon Eclipse 80i microscope using either brightfield or fluorescent optics. Photomicrographs were produced with a Nikon digital camera attached to the microscope. Qualitative estimates of antibody binding in specific brain sites were made by considering signal strength and regional boundaries. An image editing software program, Adobe Photoshop 7.0 (San Jose, CA), was used to adjust contrast and brightness.

### Iodination of TAM-163 and Preparation of the Dosing Solution for the Biodistribution Study

Iodination was performed by the IODO-BEADS method according to manufacturer’s instructions (Pierce, Rockford, IL, USA), using ∼0.5 mg of TAM-163 re-formulated in phosphate buffer saline (PBS) and 5 mCi of ^125^-Iodine (Perkin Elmer; Waltham, MA, USA). A dosing solution was prepared by mixing unlabeled TAM-163, a trace amount of ^125^I-labeled TAM-163, and PBS to achieve the specific activity of ∼84 µCi/mg (with ∼5.6% free iodine) and the final protein concentration of 0.75 mg/mL. Dosing solution was characterized by gamma-counting of trichloroacetic acid (TCA)-precipitable radioactivity and gel electrophoresis, as previously described [22].

For the biodistribution study with ^125^I-TAM-163, six-week-old male lean C57BL/6J mice were given water containing potassium iodide (0.1 mg/mL) for 3 days prior to dosing and the dosing solution was administered as a single IV bolus dose into the tail vein (at a volume of 4 mL/kg to resulting in a 1 mg/kg dosage). Animals were deeply anesthetized with an IP injection of ketamine:xylazine (100∶20 mg/kg, IP) and subsequently perfused transcardially with heparinized (25 U/mL) PBS. Serum (50–100 µL) and weighed tissue samples were taken at 1 hour and then at 1, 2, 6, 13, 20, 28, and 50 days post dose, with six mice per time point. Radioactive equivalent (RE) concentrations in serum and tissues were determined by gamma-counting based on TCA-precipitable and total counts, respectively, as described previously [22].

### Immunoassay for Determination of TAM-163 Concentrations in Serum Samples

TAM-163 antibody in serum samples was captured onto a microtiter plate pre-coated with rhTrkB- ECD. The bound TAM-163 antibody was detected with a mouse anti-human IgG monoclonal antibody conjugated to horseradish peroxidase (HRP). The enzyme substrate, 3, 3′, 5,5′-tetramethylbenzidine (TMB), was used to produce a colored end-product. Optical densities (OD) were measured using a microplate spectrophotometer at 450 nm. Sample concentrations were determined by interpolation from a calibration curve that was fit using a 4 parameter logistic equation (Softmax Pro, version 4.3.1, Molecular Devices and Watson LIMS, version 7.0.0.01, Thermo Electron Corporation). The lower limit of quantitation (LLOQ) was 14.6 ng/mL.

### Statistical Analysis

Data is expressed as means ± s.e.m. Statistical significance between two groups was assessed by one-way ANOVA followed by Tukey’s multiple comparison test. Comparisons of multiple groups over time were performed by repeated measures ANOVA followed by Tukey’s multiple comparison test. A *p* value of less than 0.05 was considered significant.

## Results

**Figure 1:**
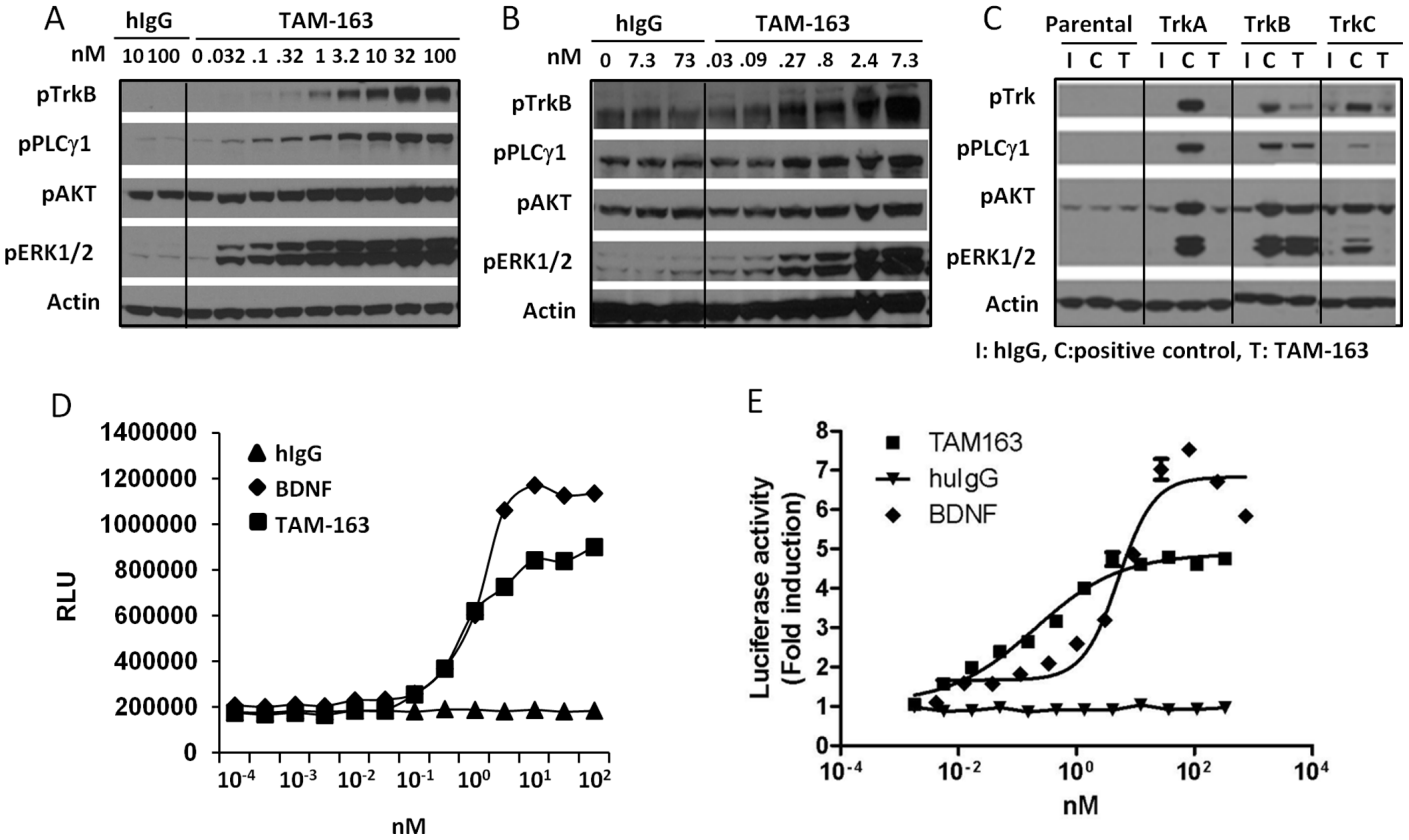
TAM-163 selectively activates recombinant human and mouse TrkB. Phosphorylation of TrkB, PLCγ1, AKT and ERK1/2 in HEK293 cells transfected with human TrkB (A), mouse TrkB (B) or human TrkA, TrkB or TrkC (C). I: hIgG, C: Positive controls (BDNF (Parental and TrkB), NGF (TrkA), NT-3 (TrkC)), T: TAM-163. Recruitment of SHC1 to hTrkB in U2OS cells (D). Luciferase reporter assay in HEK293 cells expressing human TrkB (E).

TrkB is a high affinity catalytic receptor for several growth factor-related proteins (neurotrophins), in particular BDNF and NT-4 [17], [23]. Binding of BDNF or NT-4 to TrkB has been shown to induce receptor dimerization, autophosphorylation and several intracellular signaling cascades, including activation of MAPK and PI3K/AKT pathways and phosphorylation of PLCγ1 [17]. We have previously described a mouse monoclonal antibody, 29D7, that is a potent and selective agonist for both human and rodent TrkB. 29D7 binds to the extracellular domain of human or mouse TrkB with high affinity, activates the downstream TrkB signaling cascade in cells, and promotes neurite outgrowth and neuronal survival in human neuroblastoma and primary rat or mouse neurons [18]. Similar to endogenous TrkB ligands, we have shown that 29D7 induces potent and sustained weight loss in diet-induced obese mice, (Manuscript in preparation). To assess the effects of TrkB agonism in other species, including non-human primates, we humanized 29D7 to generate TAM-163. Surface plasmon resonance showed that TAM-163 binds to the purified recombinant extracellular domain of both human and mouse TrkB with an affinity similar to 29D7 (Table 1). In cells over-expressing recombinant human (Figure 1A) or mouse (Figure 1B) TrkB, TAM-163 activated multiple arms of the TrkB signaling cascade. In particular, TAM-163 dose-dependently increased TrkB autophosphorylation and phosphorylation of PLCγ1, AKT and ERK1/2. Importantly, binding and signaling by TAM-163 were specific to TrkB and no cross-reactivity with TrkA or TrkC was observed in direct binding assays (data not shown) or signaling assays (Figure 1C). Furthermore, similar to 29D7, no binding of TAM-163 to p75NTR was observed (data not shown). To quantitate the effects of TAM-163 on signaling and compare its potency to the endogenous ligand BDNF, we used two high throughput assays in cells over-expressing recombinant human TrkB. In both assays, TAM-163 potently activated TrkB-mediated signaling as shown by increased recruitment of SHC1 to human TrkB in U2OS cells (Figure 1D), and increased transcriptional activation of a CRE-reporter construct (Figure 1E). While the potency of TAM-163 on SHC1 recruitment appeared comparable to BDNF (EC_50_s of 0.67 nM and 1 nM for TAM-163 and BDNF, respectively), TAM-163 was more potent than BDNF in the CRE-reporter assay (EC_50_s of 0.37 nM and 5.2 nM for TAM-163 and BDNF, respectively). Interestingly, the maximal efficacy achieved by TAM-163 was less than achieved by BDNF in both assays, suggesting that TAM-163 is a partial TrkB agonist.

**Table 1:** TAM-163 binds to recombinant human and mouse TrkB.

Ligand 1	Ligand 2	KD (nM)	Ka	kd
TAM-163	hTrkB	9.4	4.3×10^5^	4.1×10^−3^
TAM-163	mTrkB	11.4	4.4×10^5^	5.0×10^−3^
29D7	hTrkB	14.7	2.1×10^5^	3.1×10^−3^
29D7	mTrkB	16.3	4.2×10^5^	6.9×10^−3^

The binding of TAM-163 and parent molecule 29D7 to the extracellular domain of human or mouse recombinant TrkB protein was measured by surface plasmon resonance. Affinity (KD), association rate constant (ka) and dissociation rate constant (kd) were calculated using a 1∶1 fit model.

**Figure 2:**
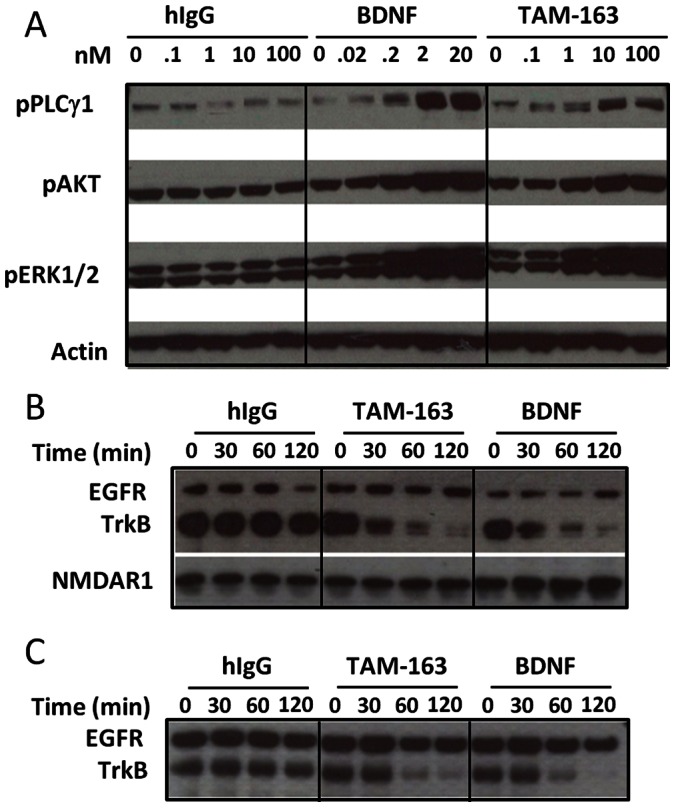
TAM-163 activates endogenous human TrkB. Phosphorylation of PLCγ1, AKT and ERK1/2 in human neuroblastoma SH-SY5Y (A). Internalization (B) and degradation (C) of hTrkB in SH-SY5Y cells.

To determine the effects of TAM-163 on endogenous TrkB activation, we next examined TAM-163-mediated signaling in the human neuroblastoma cell line SH-SY5Y. This cell line, upon differentiation, expresses endogenous TrkB mRNA at levels similar to what is found in human brain (G. Feng, unpublished data). TAM-163 increased phosphorylation of PLCγ1, AKT and ERK1/2 with a potency comparable to BDNF, but similar to what was observed in recombinant cell lines, TAM-163 appeared to act as a partial agonist (Figure 2A). An important aspect of TrkB signaling is ligand-induced internalization and degradation of TrkB. To investigate these processes, we used cell surface biotinylation either after activation to determine the amount of TrkB remaining on the cell-surface (internalization assay) or prior to activation to follow the fate of cell-surface TrkB (degradation assay). As can be seen in Figures 2B and C, TAM-163 induced internalization and degradation of TrkB in SH-SY5Y cells in a manner similar to BDNF, suggesting that intracellular trafficking of TrkB upon activation by TAM-163 is similar to what is observed with the endogenous ligand BDNF.

To determine the potential of TAM-163 to cross-react with TrkB from several species, we obtained the cDNA sequences from rats, hamsters, dogs, and cynomolgus monkeys. While we did not formally test the ability of TAM-163 to activate rat and hamster TrkB receptors, the high degree of identity between mouse, rat and hamster TrkB extracellular domains (Figure S1) together with the efficacy of TAM-163 in vivo in rats and hamsters (see below) strongly suggest that TAM-163 retained the cross-reactivity to other rodent species. In stable cell lines over-expressing dog TrkB, we observed that both BDNF and TAM-163 increased pTrkB, pPLCγ1 and pERK1/2 in a dose-dependent manner (Figure S2), confirming the cross-reactivity of TAM-163 with this species. Finally, because the extracellular domain of both cynomolgus (Figure S1) and rhesus monkey (not shown) TrkB showed 100% amino acid identity with human TrkB, TAM-163 is expected to interact with monkey TrkB in a manner similar to human TrkB.

**Figure 3:**
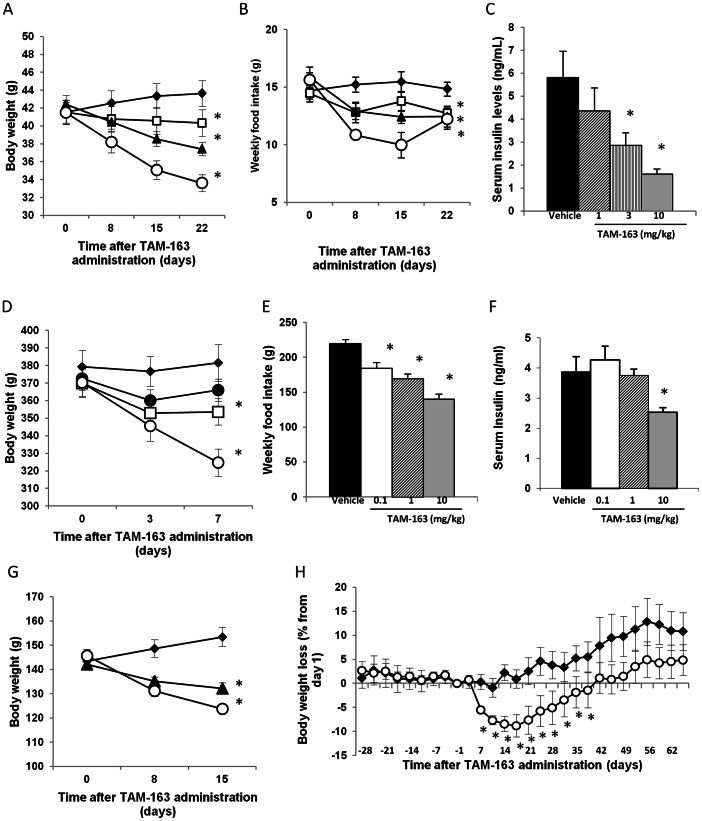
TAM-163 induces body weight loss and appetite suppression in several animal species. Body weight, food intake and serum insulin levels in diet-induced obese (DIO) mice (A–C) or Zucker Diabetic Fatty (ZDF) rats (D–F) (n = 8–10 per group). Body weight of Syrian gold hamsters fed a high-fat high-cholesterol diet plus 10% sucrose in drinking water for 2 weeks prior to study initiation (G) (n = 9 per group). Body weight of chow fed adult beagle dogs (H) (n = 4 for vehicle and n = 8 for TAM-163-treated dogs). Vehicle: filled diamonds, TAM-163 0.1 mg/kg: filled circles, TAM-163 1 mg/kg: open squares, TAM-163 3 mg/kg: filled triangles, TAM-163 10 mg/kg: open circles. Data is shown as mean ± SEM, *p<0.05 when comparing TAM-163-treated animals with vehicle-treated animals.

To examine the effects of TAM-163 *in vivo*, we first used diet-induced obese (DIO) mice since both TrkB endogenous ligands, BDNF and NT-4, as well as the TAM-163 parent molecule 29D7 potently decreased body weight and food intake in this model (Refs 7 and 9, and manuscript in preparation). As expected, TAM-163 dose-dependently decreased body weight in DIO mice over a period of 3 weeks with a maximal effect of approximately 20% at 10 mg/kg (Figure 3A). This decrease was accompanied by a concomitant reduction in food intake (Figure 3B) and dose-dependent improvement in insulin levels (Figure 3C).

Similarly to what we observed in mice, TAM-163 dose-dependently decreased body weight in obese and diabetic ZDF rats by approximately 12% at 10 mg/kg after one week of treatment, with concomitant reduction in food intake (Figures 3D and E). These changes were accompanied by a significant improvement in insulin levels at the 10 mg/kg dose (Figure 3F). To further assess the efficacy of TAM-163 in other species, body weight and food intake were monitored in hamsters that were dosed weekly for 2 weeks and in dogs that received a single TAM-163 dose. TAM-163 induced significant weight loss in both models (Figures 3G and H) and caused appetite suppression in dogs (not shown), suggesting that activation of TrkB receptor is an important negative regulator of body weight homeostasis and satiety in both rodents and some non-rodent species.

**Figure 4:**
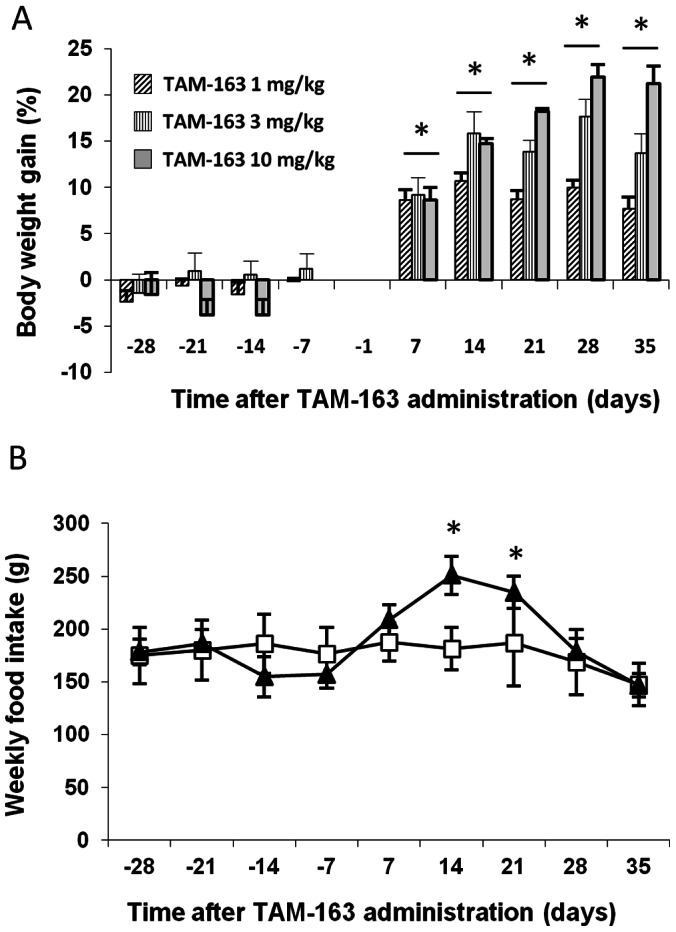
Single dose TAM-163 induces body weight gain and increases food intake in high-fat fed obese rhesus monkeys. Body weight (A) and food intake (B) of male rhesus monkeys fed a high-fat diet for 9 months. Animals received a single dose of vehicle (his-sucrose) or TAM-163 (1, 3 or 10 mg/kg) and were followed for a total of 35 days. Data is shown as mean ± SEM, *p<0.05 as compared to pre-TAM-163 administration. (n = 5 per group). TAM-163 1 mg/kg: open squares, TAM-163 3 mg/kg: filled triangles.

**Figure 5:**
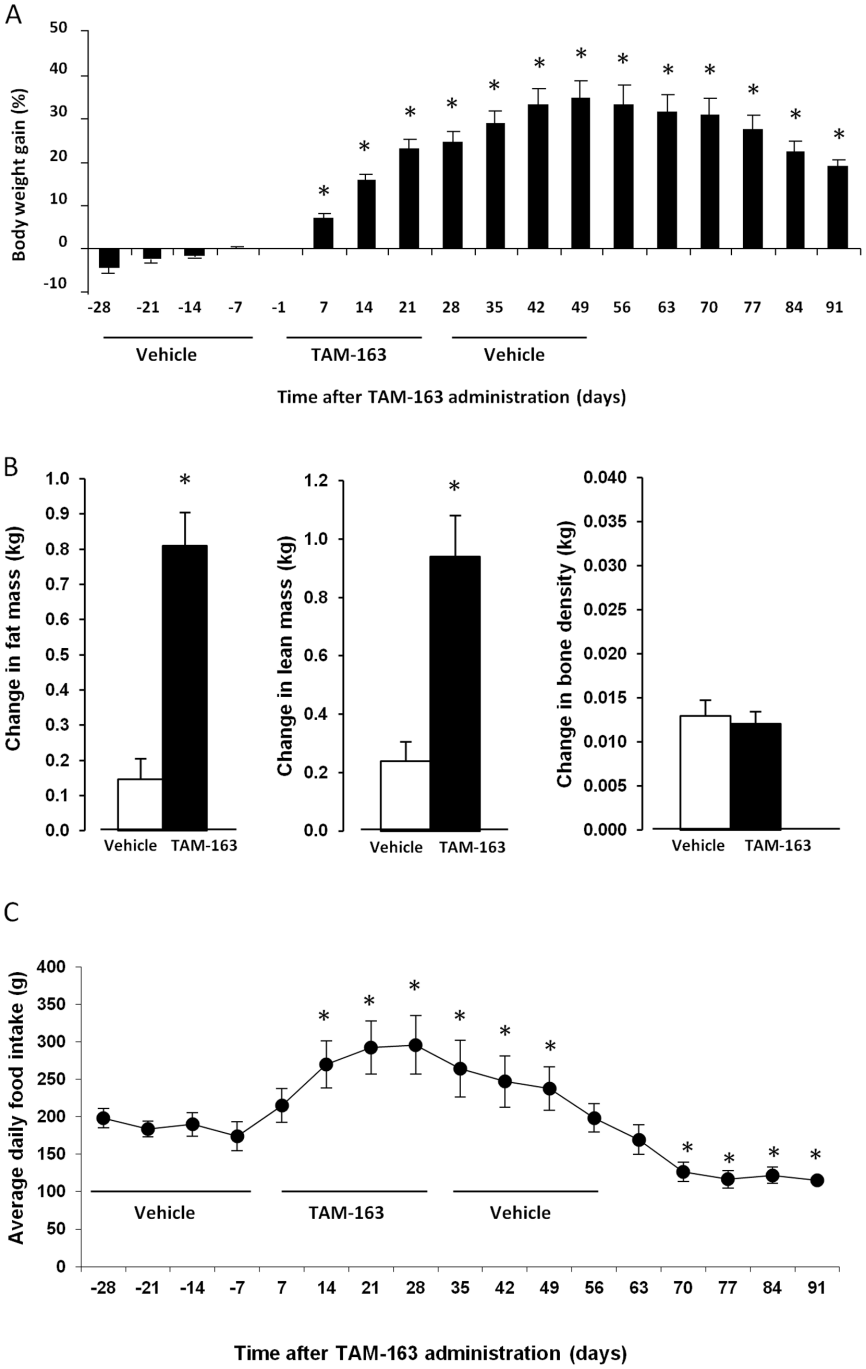
Repeated dosing of TAM-163 induces body weight gain and increases food intake in high-fat fed obese rhesus monkeys. Body weight (A), body composition (B) and food intake (C) of male rhesus monkeys fed a high-fat diet for 9 months. Animals received four weekly doses of vehicle (his-sucrose), 4 weekly doses of TAM-163 (10 mg/kg) and were washed-out with 4 weekly doses of vehicle. Body weight and food intake were recorded for a total of 91 days. Body composition was performed after the last dose of vehicle (∼day 25) and TAM-163 (∼day 53). Data is shown as mean ± SEM, *p<0.05 as compared to pre-TAM-163 administration. (n = 10).

Previous studies have shown that peripheral administration of the endogenous TrkB ligand NT-4 increased body weight and food intake in lean cynomolgus, lean rhesus and obese baboon monkeys [7]. To determine the effects of TAM-163 in an obese monkey model comparable to our DIO mice, we used diet-induced obese rhesus monkeys. Following acclimation to experimental conditions, animals were treated for 4 weeks with vehicle followed by 1 or 4 weeks of TAM-163 and subsequent washout or a second vehicle period, using a crossover design. At the beginning of the experiment, monkeys weighed an average of 7 kg (range: 5.3 kg to 10.5 kg) and gained approximately 4% of their body weight during the 4 week vehicle run-in period (Figures 4A and 5A), which was considered their background weight gain. After a single injection, TAM-163 significantly increased body weight gain in a dose-dependent manner with a maximum increase of 10, 18 and 22% reached 28 days after a single IV injection of 1, 3 or 10 mg/kg (Figure 4A). The increased weight gain was accompanied by increased food intake at 14 and 21 days post-TAM-163 injection at the 3 mg/kg dose (Figure 4B).

We next examined the effects of repeat dosing of TAM-163 in obese rhesus monkeys. After a 4-week vehicle run-in period, monkeys received four weekly injections of TAM-163 (10 mg/kg) followed by a washout period consisting of four weekly injections of vehicle (Figure 5A). TAM-163 administration significantly increased body weight gain with a maximal increase of 35% 49 days following the initiation of the treatment period (Figure 5A). Body weight gain subsided from that point on, but 91 days following the first TAM-163 injection, animals were still 20% heavier compared to their pre-TAM-163 body weight (Figure 5A). Serum chemistry, hematology, daily clinical observations, and behavioral assessments showed no signs of fluid retention and no evidence of abnormal behavior, while body composition analysis showed that fat and lean masses contributed equally to the body weight gain induced by TAM-163 (Figure 5B). Concomitant with the body weight gain observed with TAM-163 treatment, a significant increase in food intake was observed from 14 up to 49 days post TAM-163 injection, with a maximum increase of 63% 28 days following the initiation of the treatment period as compared to pre-treatment (day-7) (Figure 5C). Interestingly, the increase in food intake abated concomitant with the reversal of body weight gain (d. 56) and food intake was significantly decreased starting at day 70 of the treatment period, potentially a feedback mechanism to restore body weight to initial measurements (Figure 5C). Importantly, the effects of TAM-163 on both body weight and food intake were reversible. Overall, this data suggest that TAM-163 is a potent appetite stimulant leading to significant body weight gain in obese rhesus monkeys.

To investigate possible reasons for differences in TAM-163 pharmacology between monkeys and rodents, we examined the relationship between body weight changes and TAM-163 circulating levels after peripheral administration of TAM-163 to either lean Sprague-Dawley rats or lean cynomolgus monkeys. Similar to what was observed in obese animals, low doses of TAM-163 caused a significant decrease in body weight gain in rats, but a significant increase in body weight gain in monkeys (Table 2). In rats, increasing doses of TAM-163 continued to decrease body weight gain, including the very high dose of 200 mg/kg (Table 2). In contrast, body weight gain was only observed at low and intermediate doses of TAM-163 (up to 60 mg/kg) in monkeys, while very high doses of TAM-163 (200 mg/kg) showed either no significant body weight gain (male) or body weight loss (female) (Table 2). The serum exposure of animals to TAM-163 was comparable between the two species and increased with dose, suggesting that the differential effects on body weight in the two species cannot be explained by different exposures to TAM-163 (Table 2).

**Table 2:** Body weight change and TAM-163 exposure following intravenous (IV) administration of TAM-163 to lean Sprague-Dawley rats and lean cynomolgus monkeys.

		Maximum change in body weight (%)		AUC (0-∞) (µg*hr/mL)	
Species	Dose (mg/kg)	Males	Females	Males	Females
**Sprague-Dawley Rats**	3	17↓		2,926	
	20^a^	23↓	14↓	42,241	46,739
	60^a^	27↓	20↓	203,558	161,849
	200^a^	37↓	17↓	664,920	637,509
**Cynomolgus monkeys**	1	5↑		2,206	
	3		5↑		
	20^b^	6↑	7↑	67,414	64,025
	60^b^	3↑	7↑	260,115	226,545
	200^b^	1↑	8↓	827,068	689,691

a Listed is the group mean maximum % change in body weight compared with vehicle controls; this is the maximum % change body weight throughout the study after administration of a single dose (n = 10/sex/group).

b For NHPs, listed is the group mean maximum % change in body weight compared with the last pretest body weight; this is the maximum % change in body weight on Day 7 after administration of a single dose (n = 6 males/group and n = 3 females/group).

**Figure 6:**
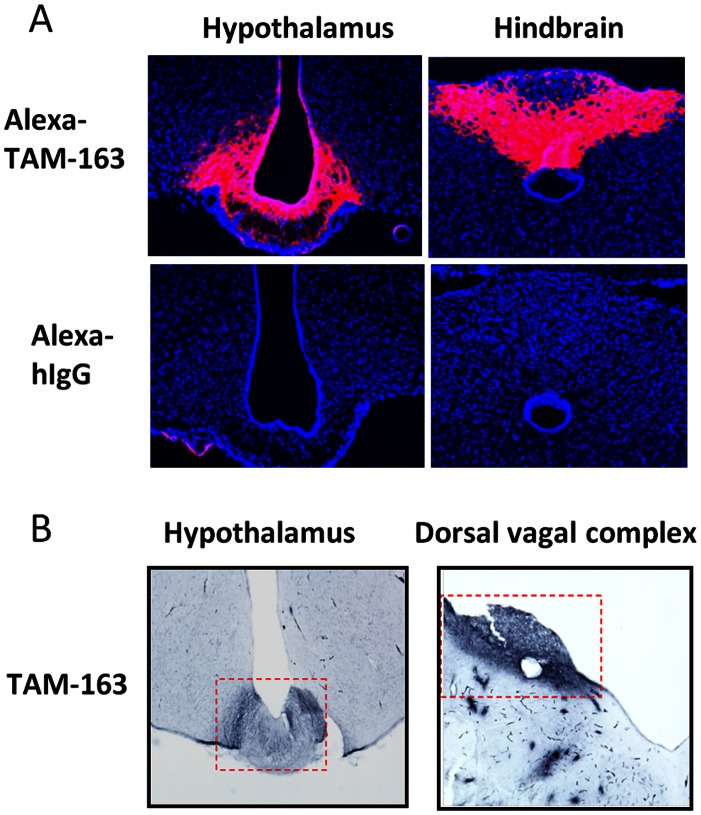
TAM-163 localizes to the mediobasal hypothalamus and dorsal-vagal complex of the brain in both mice and monkeys. A single dose of Alexa-labeled TAM-163 was injected to mice (10 mg/kg, IP) or monkeys (10 mg/kg, IV) and detected in the hypothalamus and dorsal vagal complex of the brain 6 hrs after administration in diet-induce obese (DIO) mice (A) or lean cynomolgus monkeys (B). (n = 3–4 per group).

To address potential differences in TAM-163 distribution to the target organ (brain), we examined the brain penetration of peripherally administered TAM-163 in rodents and monkeys. First, we determined the tissue distribution profile of ^125^I-labeled TAM-163 in lean mice. The relative concentrations of TAM-163 were highest in the serum and mean tissue-to-serum (T/S) ratios were <25% for all tissues and time-points examined with average T/S ratios <10% for most tissues. As expected from an antibody, average T/S ratios in the brain ranged from 0.3 to 0.6% over the 50 day period post a single dose (Table S1). Next, we examined TAM-163 distribution in appetite-regulatory centers of the brain by visualizing fluorescently-labeled TAM-163 (Alexa-TAM-163) six hours after peripheral injection in DIO mice and lean monkeys. All Alexa-TAM-163-treated mice showed a robust fluorescent signal in regions of the dorsal-vagal complex in the hindbrain as well as in the mediobasal hypothalamus, including portions of the arcuate nucleus and the median eminence (Figure 6A). These areas are known to form part of the circumventricular organ and have been implicated in appetite regulation [14]. No signal was observed in other areas of the hypothalamus, or in any other brain regions (data not shown), and no signal was observed in the hypothalamus or the hindbrain of mice injected with control Alexa-IgG (Figure 6A). Due to high autofluorescence, we were unable to use fluorescent imaging in monkeys. However, immunohistochemistry with an anti-human IgG antibody that does not cross-react with monkey IgG was able to clearly detect peripherally injected TAM-163 in the median eminence/arcuate nucleus of the hypothalamus as well as in the dorsal-vagal complex of the hindbrain, a pattern similar to what was observed in mice (Figure 6B). Again, no staining was observed in other brain regions (data not shown) or in brains from monkeys injected with Alexa-IgG control (Figure 6B). Taken together, our data demonstrate that TAM-163 is exclusively enriched in areas of the hypothalamus and brainstem located outside the blood brain barrier and argue against the possibility that the different pharmacology of TAM-163 between rodents and monkeys is due to differences in brain penetration.

## Discussion

In this study, we examined the effects of a well-characterized TrkB agonist antibody across a range of species. TrkB and its endogenous ligand BDNF are one of only three receptor-ligand pairs (the others being leptin/leptin-receptor and MC4/MC4 receptor) that have profound effects on body weight and food intake in mice and in man, as demonstrated in genetic studies. Thus, it is tantalizing to speculate that pharmacological modulation of this pathway could provide potent and effective therapies for body weight disorders, such as obesity. Efforts to exploit this pathway to date, however, have been disappointing and confusing. Initial studies using the endogenous TrkB ligand BDNF showed potent weight loss in a variety of rodent animal models [7], [9], [10]. However, while BDNF has been examined in numerous clinical studies ranging from single-dose to 9-month chronic administration in normal human volunteers, patients with diabetic neuropathy or patients with amyotrophic lateral sclerosis, no effects of BDNF on food intake or body weight have been reported, and no studies were conducted to directly evaluate such effects [24], [25]. More recently, a second TrkB ligand, NT-4, was found to cause potent weight loss in rodents, but equally potent weight gain in non-human primates [7], [26]. Studies comparing peripheral and central injection of NT-4 suggested that in non-human primates, NT-4 engages both appetite-stimulating and appetite-reducing pathways, while in rodents only appetite-reducing effects of NT-4 were identified [7]. No clinical data on NT-4 have been reported to date, and no data are available on the effects of peripherally injected BDNF in non-human primates. A TrkB-specific mouse monoclonal agonist antibody, 38B8, recapitulates the effects of peripherally administered NT-4 on body weight in mice and lean cynomolgus monkeys, suggesting that these effects of NT-4 are mediated by TrkB. However, 38B8 has not been extensively characterized in vivo and its effects in man are unknown [7].

Here we use a well-characterized monoclonal TrkB agonist antibody, TAM-163, to examine the pharmacological effects of peripheral TrkB activation in different species. Unlike 38B8, TAM-163 contains a human IgG backbone, which increases its half-life and decreases its immunogenicity in primates, while retaining its properties in rodents, making it a more suitable tool reagent for non-human primates. The parent molecule of TAM-163, 29D7, has been well characterized both in vitro and in vivo [18], and we have shown that TAM-163 retains binding and signaling properties of 29D7 across multiple species. TAM-163 activates TrkB and induces its internalization and degradation in a manner very similar to BDNF, suggesting similar intracellular trafficking after receptor activation. One major difference between TAM-163 and BDNF is that TAM-163 appears to be a partial agonist with respect to several signaling pathways. Since this partial agonism is present to a similar extent for both mouse and human TrkB, comparison across species is still valid using this reagent.

The preclinical *in vivo* pharmacology of TAM-163 is similar to what has been described for the endogenous TrkB ligands BDNF and NT-4. We observe potent weight loss in mice, but equally potent weight gain in monkeys. Our data extend previous preclinical studies with the TrkB-specific reagent 38B8 in several ways. First, we demonstrate that weight loss with a TrkB-specific agonist is observed not only in mice, but also in rats, hamsters and dogs. Second, we show that weight gain induced by a TrkB-specific agonist is present in lean cynomolgus and obese rhesus monkeys and is reversible in both species. Careful *in vitro* signaling experiments as well as cloning and examination of the TrkB sequences from several species establish that the observed effects are unlikely to be linked to differential activation of TrkB across species. Finally, we show that the opposite pharmacology observed with TAM-163 between rodents and non-human primates is observed primarily at low doses of a peripherally administered TrkB agonist, and cannot be explained by differential exposure to the antibody in the different species.

An important aspect of our study is the comparison of pharmacological effect with exposure in rodents and monkeys at different doses of TAM-163. Based on central and peripheral injection experiments of NT-4, a dual role of TrkB in the regulation of food intake in monkeys has been suggested, where activation of peripheral TrkB increases energy intake, while central activation of TrkB decreases energy intake [7]. The biodistribution parameters of TAM-163 in mice are in line with those observed with other human IgGs, with tissue-to-serum concentration ratios of ∼10–20% in perfused organs and 0.1–0.6% in brain [22]. Although we have not been able to study the biodistribution of radiolabeled TAM-163 in monkeys, it is reasonable to assume similar values for primates. We hypothesize that low doses of TAM-163 (1–20 mg/kg) result in exposures sufficient to reach target engagement in peripheral tissues and CVOs, but not in deeper brain structures (inside BBB), leading to the activation of a subset of neurons that may not be critical for appetite suppression. However, at high doses (100–200 mg/kg), the exposure of TAM-163 would lead to target engagement in peripheral tissues, CVOs and deeper brain structures. We thus propose that the lack of weight gain observed in non-human primates at high doses of TAM-163 reflects engagement of the TrkB receptor both inside (central) and outside the BBB (peripheral). It would appear in this case that appetite-stimulating and appetite-reducing circuits cancel each other out, although some weight loss is observed in female monkeys at the highest doses. Thus, our data validate the “dual role” hypothesis for TrkB effects on appetite in monkeys.

The molecular mechanism responsible for the paradoxical differences between rodents and non-human primates is currently unknown, but we speculate that the neurocircuitry involved in food intake regulation may differ between these two species. Precedent for such species-differences has been reported for other appetite-regulatory circuits. For example, it has been reported that NPY is mainly expressed in the arcuate nucleus of the hypothalamus in adult rats, while it is also found in several hypothalamic regions in non-human primates, including paraventricular nucleus and supraoptic nucleus [27]. Furthermore, the anatomy of CART-containing neurons in humans was found to differ markedly from that observed in rodent brain, with CART expression found in NPY/AGRP neurons in humans but not in rodents [28]. It is thus possible that, although TAM-163 reaches similar regions of the brain in rodents and non-human primates, the neuroanatomy and/or neuronal projections differ significantly between species, leading to the recruitment of different downstream effectors involved in the regulation of energy balance.

In summary, our data show that TrkB activation with low doses of a humanized antibody leads to significant weight loss in several species, including mice, rats, hamsters and dogs, but significant weight gain in non-human primates. Circulating drug levels and brain localization of TAM-163 could not explain these differences, suggesting a possible difference in brain neuroanatomy between rodents and monkeys. Our data confirm previous reports of species-differences in response to TrkB agonist ligands. Clinical evaluation of TrkB agonists will be required to determine the pharmacology of this pathway in man and the potential utility of TrkB agonist agents for body-weight regulatory disorders, such as obesity, anorexia or cachexia.

## Supporting Information

Figure S1 **TrkB sequences across species.** (A) Amino acid sequences of human, cynomolgus monkey, dog, hamster, mouse and rat TrkB. Signal sequence and extracellular domain are indicated by single and double underlines. (B) % identity between the extracellular domains of TrkB from different species.
(TIF)

Figure S2 **TAM-163 activates downstream signaling of TrkB in dogs.** Phosphorylation of TrkB, PLCγ1, AKT and ERK1/2 in HEK293 cells transfected with dog TrkB.
(TIF)

Table S1 Mean (± SD) tissue to serum (T/S) radioactive equivalent concentration ratio after a single IV dose of 3 mg/kg of [^125^I]TAM-163 to lean male C57BL/6 mice.
(DOC)

## References

[1] Gray J, Yeo GSH, Cox JJ, Morton J, Adlam AR, et al (2006) Hyperphagia, severe obesity, impaired cognitive function, and hyperactivity associated with functional loss of one copy of the brain-derived neurotrophic factor (BDNF) gene. Diabetes 55: 3366–3371. 17130481 10.2337/db06-0550 PMC2413291

[2] Han JC, Liu QR, Jones M, Levinn RL, Menzie CM, et al (2008) Brain-derived neurotrophic factor and obesity in the WAGR syndrome. N Engl J Med 359: 918–927. 18753648 10.1056/NEJMoa0801119 PMC2553704

[3] Rios M, Fan G, Fekete C, Kelly J, Bates B, et al (2001) Conditional deletion of brain-derived neurotrophic factor in the postnatal brain leads to obesity and hyperactivity. Mol Endocrinol 15: 1748–1757. 11579207 10.1210/mend.15.10.0706

[4] Xu B, Goulding EH, Zang K, Cepoi D, Cone RD, et al (2003) Brain-derived neurotrophic factor regulates energy balance downstream of melanocortin-4 receptor. Nat Neurosci 6: 736–742. 12796784 10.1038/nn1073 PMC2710100

[5] Yeo GS, Connie Hung CC, Rochford J, Keogh J, Gray J, et al (2004) A de novo mutation affecting human TrkB associated with severe obesity and developmental delay. Nat Neurosci 7: 1187–1189. 15494731 10.1038/nn1336

[6] Unger TJ, Calderon GA, Bradley LC, Sena-Esteves M, Rios M (2007) Selective deletion of Bdnf in the ventromedial and dorsomedial hypothalamus of adult mice results in hyperphagic behavior and obesity. J Neurosci 27: 14265–14274. 18160634 10.1523/JNEUROSCI.3308-07.2007 PMC6673437

[7] Lin JC, Tsao D, Barras P, Bastarrachea RA, Boyd B, et al (2008) Appetite enhancement and weight gain by peripheral administration of TrkB agonists in non-human primates. PLoS ONE 3: e1900. 18382675 10.1371/journal.pone.0001900 PMC2270901

[8] Wang C, Bomberg E, Billington C, Levine A, Kotz CM (2007) Brain-derived neurotrophic factor in the hypothalamic paraventricular nucleus reduces energy intake. Am J Physiol Regul Integr Comp Physiol 293: R1003–1012. 17581841 10.1152/ajpregu.00011.2007

[9] Nakagawa T, Ogawa Y, Ebihara K, Yamanaka M, Taiji M, et al (2003) Anti-obesity and anti-diabetic effects of brain-derived neurotrophic factor in rodent models of leptin resistance. International Journal of Obesity 27: 557–565. 12704399 10.1038/sj.ijo.0802265

[10] Nakagawa T, Ono-Kishino M, Sugaru E, Yamanaka M, Taiji M, et al (2002) Brain-derived neurotrophic factor (BDNF) regulates glucose and energy metabolism in diabetic mice. Diabetes Metab Res Rev 18: 185–191. 12112936 10.1002/dmrr.290

[11] Lebrun B, Bariohay B, Moyse E, Jean A (2006) Brain-derived neurotrophic factor (BDNF) and food intake regulation: a minireview. Auton Neurosci 126–127: 30–38. 10.1016/j.autneu.2006.02.027 16632412

[12] Muragaki Y, Timothy N, Leight S, Hempstead BL, Chao MV, et al (1995) Expression of trk receptors in the developing and adult human central and peripheral nervous system. J Comp Neurol 356: 387–397. 7642800 10.1002/cne.903560306

[13] Shibayama E, Koizumi H (1996) Cellular localization of the Trk neurotrophin receptor family in human non-neuronal tissues. Am J Pathol 148: 1807–1818. 8669468 PMC1861661

[14] Ganong W (2000) Circumventricular Organs: Definition And Role In The Regulation Of Endocrine And Autonomic Function. Clinical and Experimental Pharmacology and Physiology 27: 422–427. 10831247 10.1046/j.1440-1681.2000.03259.x

[15] Picha KM, Cunningham MR, Drucker DJ, Mathur A, Ort T, et al (2008) Protein Engineering Strategies for Sustained Glucagon-Like Peptide-1 Receptor–Dependent Control of Glucose Homeostasis. Diabetes 57: 1925–1934. 10.2337/db07-1775 PMC2453616 18426860

[16] Sun HD, Malabunga M, Tonra JR, DiRenzo R, Carrick FE, et al (2007) Monoclonal antibody antagonists of hypothalamic FGFR1 cause potent but reversible hypophagia and weight loss in rodents and monkeys. American Journal of Physiology Endocrinology and Metabolism 292: E964–E976. 17132826 10.1152/ajpendo.00089.2006

[17] Reichardt L (2006) Neurotrophin-regulated signaling pathways. Philos Trans R Soc Lond B Biol Sci 361: 1545–1564. 16939974 10.1098/rstb.2006.1894 PMC1664664

[18] Qian M, Zhang J, Tan X-Y, Wood A, Gill D, et al (2006) Novel Agonist Monoclonal Antibodies Activate TrkB Receptors and Demonstrate Potent Neurotrophic Activities. The journal of Neuroscience 26: 9394–9403. 16971523 10.1523/JNEUROSCI.1118-06.2006 PMC6674613

[19] Zhang J, Chen D, Gong X, Ling H, Zhang G, et al. (2006–2007) Cyclic-AMP response element-based signaling assays for characterization of Trk family tyrosine kinases modulators. Neurosignals 15: 26–39. 16837782 10.1159/000094385

[20] Zheng J, Shen WH, Lu TJ, Zhou Y, Chen Q, et al (2008) Clathrin-dependent endocytosis is required for TrkB dependent Akt-mediated neuronal protection and dendritic growth. J Biol Chem 283: 13280–13288. 18353779 10.1074/jbc.M709930200

[21] Chen Z-Y, Leraci A, Tanowitz M, Lee FS (2005) A Novel Endocytic Recycling Signal Distinguishes Biological Responses of Trk Neurotrophin Receptors. Mol Cell Biol 16: 5761–5772. 10.1091/mbc.E05-07-0651 PMC1289419 16207814

[22] Vugmeyster Y, Defranco D, Szklut P, Wang Q, Xu X (2010) Biodistribution of [125I]-labeled therapeutic proteins: Application in protein drug development beyond oncology. J Pharm Sci 99: 1028–1045. 19569059 10.1002/jps.21855

[23] Allen S, Dawbarn D (2006) Clinical relevance of the neurotrophins and their receptors. Clin Sci (Lond) 110: 175–191. 16411894 10.1042/CS20050161

[24] The BDNF Study Group (1999) A controlled trial of recombinant methionyl human BDNF in ALS. Neurology 52: 1427. 10227630 10.1212/wnl.52.7.1427

[25] Wellmer A, Misra VP, Sharief MK, Kopelman PG, Anand P (2001) A double-blind placebo-controlled clinical trial of recombinant human brain-derived neurotrophic factor (rhBDNF) in diabetic polyneuropathy. J Peripher Nerv Syst 6: 204–210. 11800042 10.1046/j.1529-8027.2001.01019.x

[26] Tsao D, Thomsen HK, Chou J, Stratton J, Hagen M, et al (2008) TrkB Agonists Ameliorate Obesity and Associated Metabolic Conditions in Mice. Endocrinology 149: 1038–1048. 18063676 10.1210/en.2007-1166

[27] Grove KL, Chen P, Koegler FH, Schiffmaker A, Smith MS, et al (2003) Fasting activates neuropeptide Y neurons in the arcuate nucleus and the paraventricular nucleus in the rhesus macaque. Mol Brain Res 113: 133–138. 12750015 10.1016/s0169-328x(03)00093-7

[28] Menyhert J, Wittman G, Lechan RM, Keller E, Liposits Z, et al (2007) Cocaine- and amphetamine-regulated transcript (CART) is colocalized with the orexigenic neuropeptide Y and agouti-related protein and absent from the anorexigenic a-melanocyte-stimulating hormone neurons in the infundibular nucleus of the human hypothalamus. Endocrinology 148: 4276–4281. 17525122 10.1210/en.2007-0390

