# Supplementary figures and images for: Activation of TrkB with TAM-163 Results in Opposite Effects on Body Weight in Rodents and Non-Human Primates
Source: PLoS One. 2013 May 20;8(5):e62616. doi: 10.1371/journal.pone.0062616 (PMC3659094; doi:10.1371/journal.pone.0062616)

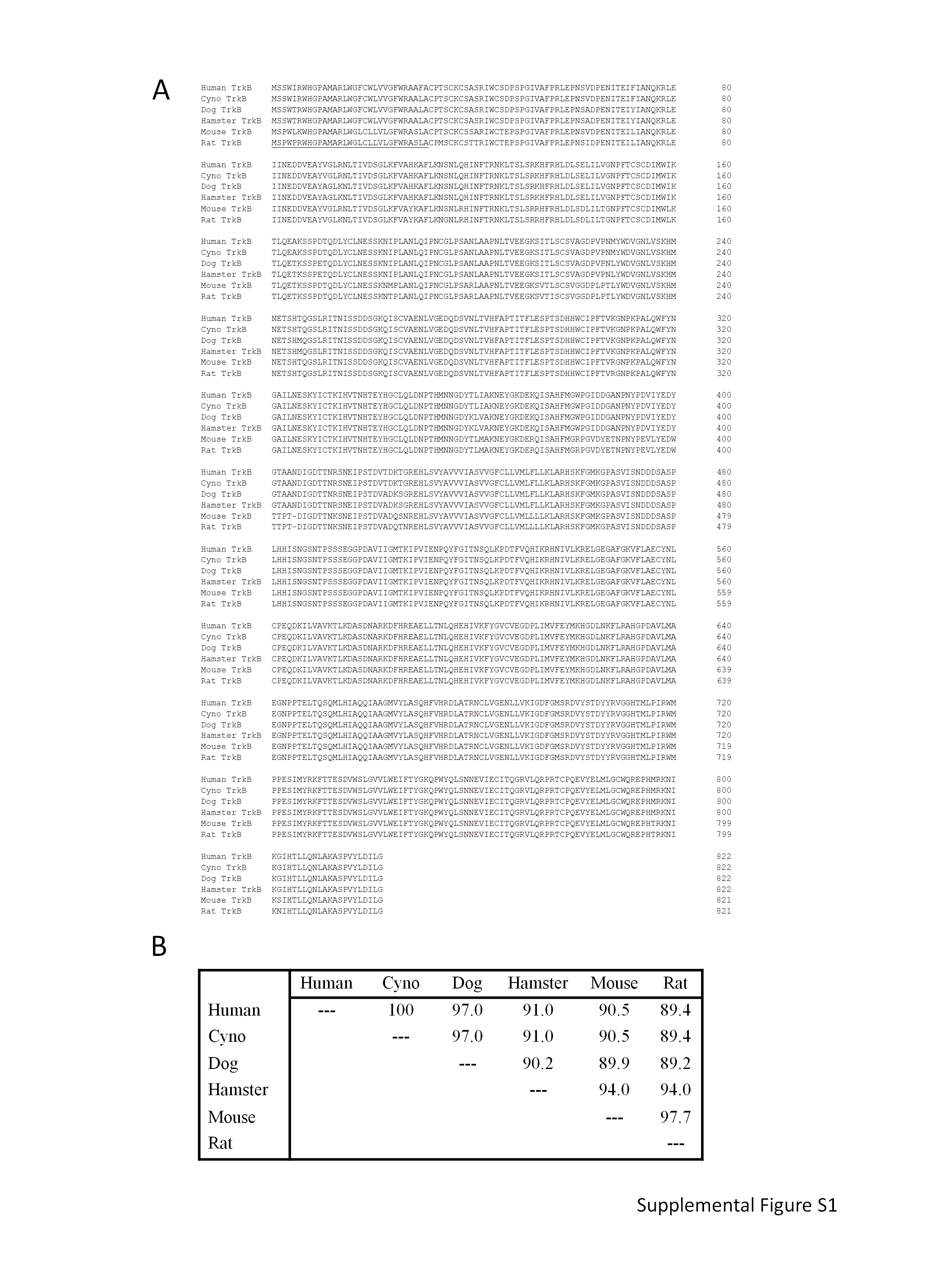

Supplement: Figure S1 — TrkB sequences across species. (A) Amino acid sequences of human, cynomolgus monkey, dog, hamster, mouse and rat TrkB. Signal sequence and extracellular domain are indicated by single and double underlines. (B) % identity between the extracellular domains of TrkB from different species. (TIF) [file pone.0062616.s001.tif]

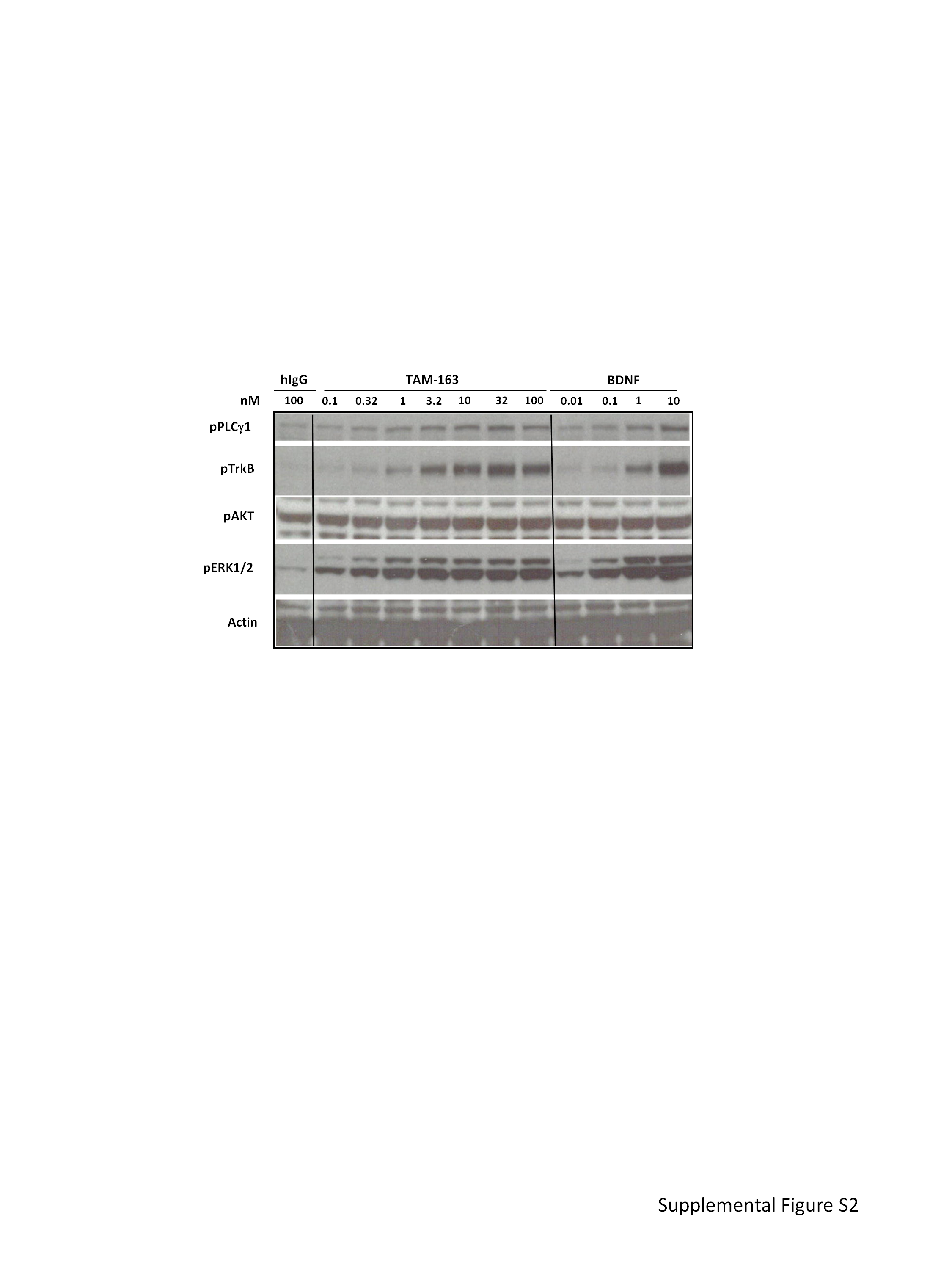

Supplement: Figure S2 — TAM-163 activates downstream signaling of TrkB in dogs. Phosphorylation of TrkB, PLCγ1, AKT and ERK1/2 in HEK293 cells transfected with dog TrkB. (TIF) [file pone.0062616.s002.tif]
