# Supplementary material for: Activation of TrkB with TAM-163 Results in Opposite Effects on Body Weight in Rodents and Non-Human Primates
Source: PLoS One. 2013 May 20;8(5):e62616. doi: 10.1371/journal.pone.0062616 (PMC3659094; doi:10.1371/journal.pone.0062616)
Supplement: Table S1 — Mean (± SD) tissue to serum (T/S) radioactive equivalent concentration ratio after a single IV dose of 3 mg/kg of [125I]TAM-163 to lean male C57BL/6 mice. (DOC) [file pone.0062616.s003.doc]

**Supplemental Table S1.** Mean (± SD) tissue to serum (T/S) radioactive equivalent concentration ratio after a single IV dose of 3 mg/kg of [^125^I]TAM-163 to lean male C57BL/6 mice.

|  | **1 hour** | **2 days** | **6 days** | **13 days** | **20 days** | **28 days** | **50 days** |
| --- | --- | --- | --- | --- | --- | --- | --- |
| **Brain** | **0.003±0.001** | **0.004±0.001** | **0.004±0.001** | **0.006±0.002** | **0.004±0.001** | **0.005±0.001** | **0.006±0.001** |
| **Colon** | **0.011±0.001** | **0.028±0.002** | **0.023±0.001** | **0.033±0.013** | **0.021±0.003** | **0.024±0.007** | **0.026±0.003** |
| **Heart** | **0.047±0.009** | **0.072±0.009** | **0.007±0.006** | **0.073±0.013** | **0.053±0.007** | **0.073±0.009** | **0.072±0.007** |
| **Kidney** | **0.040±0.010** | **0.034±0.014** | **0.026±0.005** | **0.057±0.035** | **0.028±0.005** | **0.044±0.016** | **0.055±0.026** |
| **Lung** | **0.096±0.049** | **0.065±0.032** | **0.120±0.065** | **0.132±0.047** | **0.151±0.088** | **0.089±0.048** | **0.213±0.134** |
| **Liver** | **0.031±0.008** | **0.027±0.018** | **0.016±0.002** | **0.037±0.021** | **0.016±0.002** | **0.024±0.009** | **0.026±0.009** |
| **Skeletal muscle** | **0.007±0.002** | **0.039±0.004** | **0.034±0.003** | **0.039±0.005** | **0.034±0.003** | **0.040±0.005** | **0.037±0.002** |
| **Small intestine** | **0.030±0.004** | **0.038±0.006** | **0.027±0.003** | **0.034±0.009** | **0.030±0.005** | **0.029±0.012** | **0.031±0.005** |
| **Spleen** | **0.072±0.012** | **0.050±0.006** | **0.049±0.009** | **0.063±0.018** | **0.049±0.005** | **0.056±0.011** | **0.089±0.017** |
